# Long-Term Stable Liposome Modified by PEG-Lipid in Natural Seawater

**DOI:** 10.1021/acsomega.3c10346

**Published:** 2024-02-22

**Authors:** Kayano Izumi, Jiajue Ji, Keiichiro Koiwai, Ryuji Kawano

**Affiliations:** † Department of Biotechnology and Life Science, Tokyo University of Agriculture and Technology, Tokyo 184-8588, Japan; ‡ Laboratory of Genome Science, Tokyo University of Marine Science and Technology, Tokyo 108-8477, Japan

## Abstract

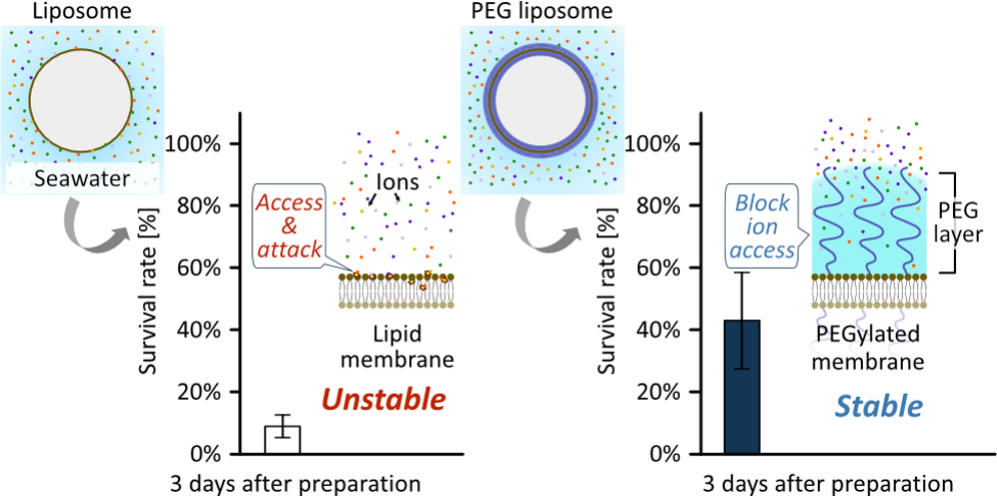

This paper describes the stabilization of liposomes using a PEGylated lipid, *N*-(methylpolyoxyethylene oxycarbonyl)-1,2-distearoyl-sn-glycero-3-phosphoethanolamine sodium salt (DSPE-PEGs), and the evaluation of the survival rate in natural seawater for future environmental applications. Liposomes in natural seawater were first monitored by confocal microscopy, and the stability was compared among different lengths and the introduction ratio of DSPE-PEGs. The survival rate increased with an increase in the PEG ratio. In addition, the survival rate in different cationic solutions (Na^+^, K^+^, Mg^2+^, and Ca^2+^ solutions) was studied to estimate the effects of the DSPE-PEG introduction. We propose that these variations in liposome stability are due to the cations, specifically the interaction between the poly(ethylene glycol) (PEG) chains and divalent ions, which contribute to making it difficult for cations to access the lipid membrane. Our studies provide insights into the use of PEG lipids and may offer a promising approach to the fabrication of liposomal molecular robots using different natural environments.

## 1 Introduction

A cell-sized liposome consisting of a lipid bilayer has been studied as a simple cell model.^1^ It has the role of a cell-like compartment for gene expression^2^ or protein production^3^ in the field of synthetic biology. Practical applications such as drug delivery have been expected in the usage of liposomes.^4,5^ It can effectively transport the drug to the diseased target by protecting the drug from degradation through encapsulation. Recently, the liposome has also attracted attention as the body of a molecular robot.^6−8^ A molecular robot is a biomolecular device consisting of sensors as perception, circuits as information processing, and actuators as vehicle functions, all integrated into a confined liposome with more focus on an engineering perspective. This robot aims to systematically control the molecular system or extend the living system rather than the artificial cells that focus on mimicking cells. The main objective of developing robots is to use them as biocompatible tools in biological environments. Chen et al. developed artificial β-cells using liposomes aiming for medical applications.^9^ The cell can sense glucose in the outer solution, drive glucose-triggered multiple molecular circuits, and release insulin-like living β-cells.

Although the applications of molecular robots have been mainly considered for use in biomedical fields, the robot also has the potential to be used in a natural environment. For example, the molecular robot will be used in natural seawater to monitor the water quality in aquaculture such as prawns.^10^ To apply the liposome in a natural environment, long-term stability is a significant problem to be solved. The stabilizing strategy of liposomes is broadly classified into three categories using nontoxic materials. (1) The gelation of the inner solution or the construction of the undercoats such as the cytoskeleton-like network has been attempted to prepare a stable liposome.^11−13^ The gel network inside the liposome forms by temperature regulation^11,12^ or light irrigation,^13^ mechanically reinforcing the liposome structure. (2) Incorporation of cholesterol into the membrane is another strategy to mechanically stabilize the liposome. Cholesterols align along lipid chains due to the amphipathic structure and form a well-packed membrane,^14^ stabilizing the bilayer configuration.^15^ (3) Covering the membrane surface with polymers such as poly(ethylene glycol) (PEG) is another strategy for stabilization as the nontoxic material. PEG is introduced to the membrane by being tagged with lipid heads and forms a shielding layer, inhibiting the adhesion of macromolecules that destroy liposomes.^16−18^

The stability of liposomes in natural seawater is still poorly understood. Natural seawater is an electrolyte aqueous solution containing a variety of components: ions, amino acids, and proteins from living organisms at the molecular level.^19−22^ To improve liposome stability, the resistance to the ions should be essential because the ions are the most abundant molecules except for H_2_O in natural seawater.^20^ Previous studies have reported that lipid bilayers interact with ions and change their physical properties.^23−27^ We consider that the interaction makes liposomes unstable, which should be solved to improve the stability.

**Figure 1:**
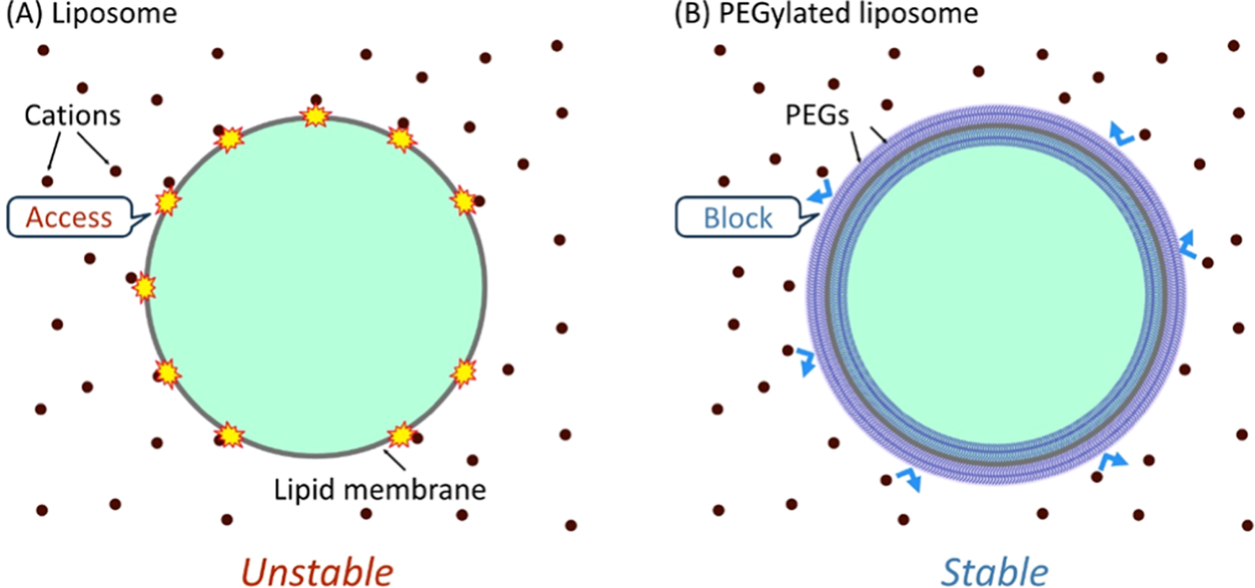
Conceptual diagram of stability improvement of the modified liposome. (A) Liposome stability was considered to be disrupted by cations and (B) to be improved by PEGs blocking the ions attacking the membrane.

We reported here the improvement of the long-term stability of liposomes by using poly(ethylene glycol) (PEG) because PEGylated lipid has the potential to inhibit the ion access to the membrane (Figure 1). We employed microscopic observation for the evaluation of the effects on the PEGs in the stabilization for more than 3 days. We first compared the liposome stability in buffer solution and natural seawater from the Miura peninsula in Japan. To evaluate the ionic effects on the membrane, we used different ionic solutions such as Na^+^, K^+^, Mg^2+^, and Ca^2+^ with Cl^–^. Finally, we considered the molecular mechanism of the ionic effects on the liposome membranes.

## 2 Experimental Section

### 2.1 Reagents

In this study, the following chemicals were used: 1-palmitoyl-2-oleoyl-glycero-3-phosphocholine (POPC; NOF corporation, Japan), 1,2-dioleoyl-*sn*-glycero-3-phospho-(1′-rac-glycerol) (sodium salt) (DOPG; Avanti Polar Lipids, AL), 1,2-dioleoyl-*sn*-glycero-3-phosphoethanolamine-*N*-(lissamine rhodamine B sulfonyl) (ammonium salt) (Rhodamine PE; Avanti Polar Lipids, AL), liquid paraffin (FUJIFILM Wako Pure Chemical Corporation, Japan), calcein (Sigma-Aldrich Co., LCC, MO), glucose (FUJIFILM Wako Pure Chemical Corporation, Japan), sucrose (FUJIFILM Wako Pure Chemical Corporation, Japan), tris(hydroxymethyl)aminomethane (Nacalai Tesque Inc., Japan), hydrochloric acid (FUJIFILM Wako Pure Chemical Corporation, Japan), sodium hydroxide (FUJIFILM Wako Pure Chemical Corporation, Japan), *N*-(methylpolyoxyethylene oxycarbonyl)-1,2-distearoyl-*sn*-glycero-3-phosphoethanolamine, 2000, sodium salt (DSPE-PEG2000; NOF Corporation, Japan), and *N*-(methylpolyoxyethylene oxycarbonyl)-1,2-distearoyl-*sn*-glycero-3-phosphoethanolamine, 5000, sodium salt (DSPE-PEG5000; NOF Corporation, Japan). 100 mM Tris-HCl was prepared from tris(hydroxymethyl) aminomethane (pH 7, adjusted with some hydrochloric acids and sodium hydroxide).

### 2.2 Preparation of Solutions

An inner solution and eight outer solutions were prepared to evaluate the liposome stability (Table 1): Natural seawater was sampled from the Miura peninsula in the Kanagawa prefecture. The seawater was filtered through a 0.45 μm filter to remove large contaminants. The inner solution was prepared so that the osmolality was the same as that of the seawater (1000 mmol/kg).^3^ Six ionic buffers containing representative seawater cations (Na^+^, K^+^, Mg^2+^, and Ca^2+^)^19,20^ were prepared to discuss the effects of cations^23−27^ on liposome stability. Chloride was used because the chloride ions have little effect on the membrane properties to neutralize the solution,^26^ and the osmolality was adjusted by glucose to be isotonic.

**Table 1:** Composition of the Inner/Outer Solution of the Liposome^a^

in/out	label	components
inner	inner buffer	1000 mM sucrose, 0.1 mM calcein, and 50 mM Tris-HCl
outer	natural seawater	natural seawater sampled at the Misaki peninsula
	buffer solution	1000 mM glucose and 50 mM Tris-HCl
	480 mM Na^+^ buffer	480 mM NaCl, 40 mM glucose, and 50 mM Tris-HCl
	10 mM K^+^ buffer	10 mM KCl, 980 mM glucose, and 50 mM Tris-HCl
	60 mM Mg^2+^ buffer	60 mM MgCl_2_, 830 mM glucose, and 50 mM Tris-HCl
	200 mM Mg^2+^ buffer	200 mM MgCl_2_, 400 mM glucose, and 50 mM Tris-HCl
	10 mM Ca^2+^ buffer	10 mM CaCl_2_, 970 mM glucose, and 50 mM Tris-HCl
	200 mM Ca^2+^ buffer	200 mM CaCl_2_, 400 mM glucose, and 50 mM Tris-HCl

a Eight solutions were prepared. The amount of inclusions in natural seawater was estimated approximately at 1000 mM (sample *N* = 8) using an osmometer (VAPRO vapor pressure osmometer Model 560, ELI Tech Group Inc.), and the osmolality between the inner and outer was adjusted by glucose to be isotonic, approximately 1000 mM (see also the Supporting Information text).

### 2.3 Preparation of Different Membrane Compositions

Polymers were used with the expectation that they would act as liposome stabilizers. They form shield-like hydrophilic layers on the surfaces and prevent protein interaction with the membrane.^16−18^ The structural difference of the PEG chains, such as length and ratio, was estimated in how it affects the stability of liposomes (see also the Supporting Information text). DSPE-PEG2000 and DSPE-PEG5000, which are specifically used in medical studies,^28−30^ were prepared to investigate the stabilizing effect of PEG length (Tables 2 and S1). In addition, the PEG mixture of DSPE-PEG2000/DSPE-PEG5000 = 1:1 (mol/mol) (DSPE-PEG2000/5000) was prepared as expected to form a bumpy hydrophilic layer and uniquely affect liposome stability. Various molar ratios of DSPE-PEG5000/POPC were also prepared to investigate the contribution of liposome stability (Tables 2 and S1).

**Table 2:** Lipid Composition of the Membranes with Different PEG Lengths or Ratios^a^

label	membrane composition*		notes
	DSPE-PEG [%]	POPC [%]	
POPC	0	100	lipid only
5 mol % DSPE-PEG2000	5	95	
5 mol % DSPE-PEG5000	5	95	
5 mol % DSPE-PEG2000/5000	total 5	95	the mixture of DSPE-PEG2000/DSPE-PEG5000 = 1:1 (mol/mol)
20 mol % DSPE-PEG5000	20	80	also prepared by fivefold lipids

a Different compositions of membranes were prepared (see also the Supporting Information text and Table S1). For each composition, + 1 mol % DOPG was further added to avoid liposomes adhering to each other and +0.1 mol % rhodamine PE was also added to recognize liposome membranes easily.

### 2.4 Preparation of Liposomes Using Droplet Transfer

Liposomes with different membrane compositions as described in Section 2.3 were prepared by using the droplet transfer method.^31^ Briefly, a 1.28 mM lipid mixture for 200 μL of chloroform was poured into a 1.5 mL centrifuge tube and settled to remove chloroform unless otherwise explained. Dried lipids were dissolved by 125 μL of liquid paraffin using a 40 kHz ultrasonic cleaner MSC-2 (AZ ONE Corporation, Japan) at 70 °C for 10 min. To form water–oil (W/O) emulsions, 25 μL of 1000 mM sucrose, 0.1 mM calcein, and 50 mM Tris-HCl dissolved in MQ (inner) were added to the tube, and the contents were mixed by tapping 40 times. 100 μL of the mixture was slowly added to 200 μL of 1000 mM glucose and 50 mM Tris-HCl which was dissolved in MQ (buffer solution) in another centrifuge tube to form a lipid monolayer at both the inner-oil and oil-outer interfaces. It was then centrifuged at 9000*g* for 20 min using a CT15E centrifuge (Hitachi Koki Co. Ltd., Japan). As the w/o emulsions transferred from the oil phase to the outer solution, a lipid bilayer membrane was formed at the interfaces, resulting in the formation of liposomes at the bottom of the tube without internal leakage. The pellet of the liposomes was extracted, and 500 μL of a buffer solution was added to the centrifuge tube. The solution was pipetted, and 250 μL of the mixture was added to a new centrifuge tube. The mixture was centrifuged again at 6000*g* for 10 min to remove small lipid aggregates. The pellet of the liposomes was extracted, and 200 μL of the outer solution (Table 1) was added to the tube.

### 2.5 Observation and Analysis of Liposome Stability

To observe the liposomes, a 3.0 mm hole was punched on a 5.0 mm-thick silicone rubber sheet and an observation chamber was made by gluing it on the cover glass. The chamber was coated with 0.1% bovine serum albumin (BSA) solution for 30 min to avoid unexpected rupture of the liposomes by attaching the chamber. After coating, 15 μL of liposome solution was added to the chamber (Figure S1A). The chamber was then sealed with another cover glass and settled for more than 30 min to sink liposomes to the bottom, and a large image of the whole chamber was taken for each measurement. An AX confocal microscope (Nikon Corp., JAPAN) equipped with a 40× water objective lens (NA 1.25, Nikon Corp., JAPAN) was used for the visualization. All experiments were performed at room temperature (RT). The liposomes were stained by rhodamine for the membrane and calcein for the inner solution. We confirmed that the liposomes were evenly distributed throughout the chamber and extracted an in-focus 1329 × 1329 μm^2^ square unit area for the analysis because liposomes distributed at the edge of the chamber were out of focus.

On the day of liposome preparation, we took the preimage and calculated liposome productivity.
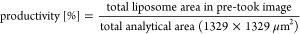
1Based on the results, we diluted the liposome concentration to occupy 5–30% of the square area by adding outer solutions. In our preliminary experiments, the fluorescents were drastically photobleached after one or two measurements. We interpreted that the fluorescents in the liposomes were completely photobleached and hardly recovered.^32^ We stored the diluted liposome solution in a 1.5 mL light-protected centrifuge tube at RT and resampled the liposomes for each measurement (Figure S1B). Liposomes were pipetted 50 times before each measurement to disperse bottom-sinking liposomes.

The stability of the liposomes was assessed by the survival rate.
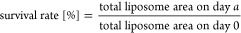
2where day *a* is the number of elapsed days defined by the preparation day as Day 0. The significant difference between the two membrane compositions was also evaluated by using the *p*-value of Welch’s *t* test. If the *p*-value was less than 0.05, then, we defined it as statistically different. Images were taken for a maximum of 14 days: the day of liposome preparation (Day 0), Day 1, Day 2, Day 3, Day 7, and Day 14. If we could not detect calcein, we confirmed whether the liposomes really disappeared or only the calcein leaked by differential interference contrast imaging or rhodamine. To better understand which factor mainly improved the liposome stability, we also examined the PEG configuration on the membrane and the size difference of the liposomes at each membrane composition (Supporting Information text). For comparison, we used the data from Day 3 unless otherwise stated, and at least three samples were prepared for each condition to ensure reproducibility.

## 3 Results

### 3.1 Stability of Liposomes with/without DSPE-PEG Evaluated in Buffer Solution and Natural Seawater

**Figure 2:**
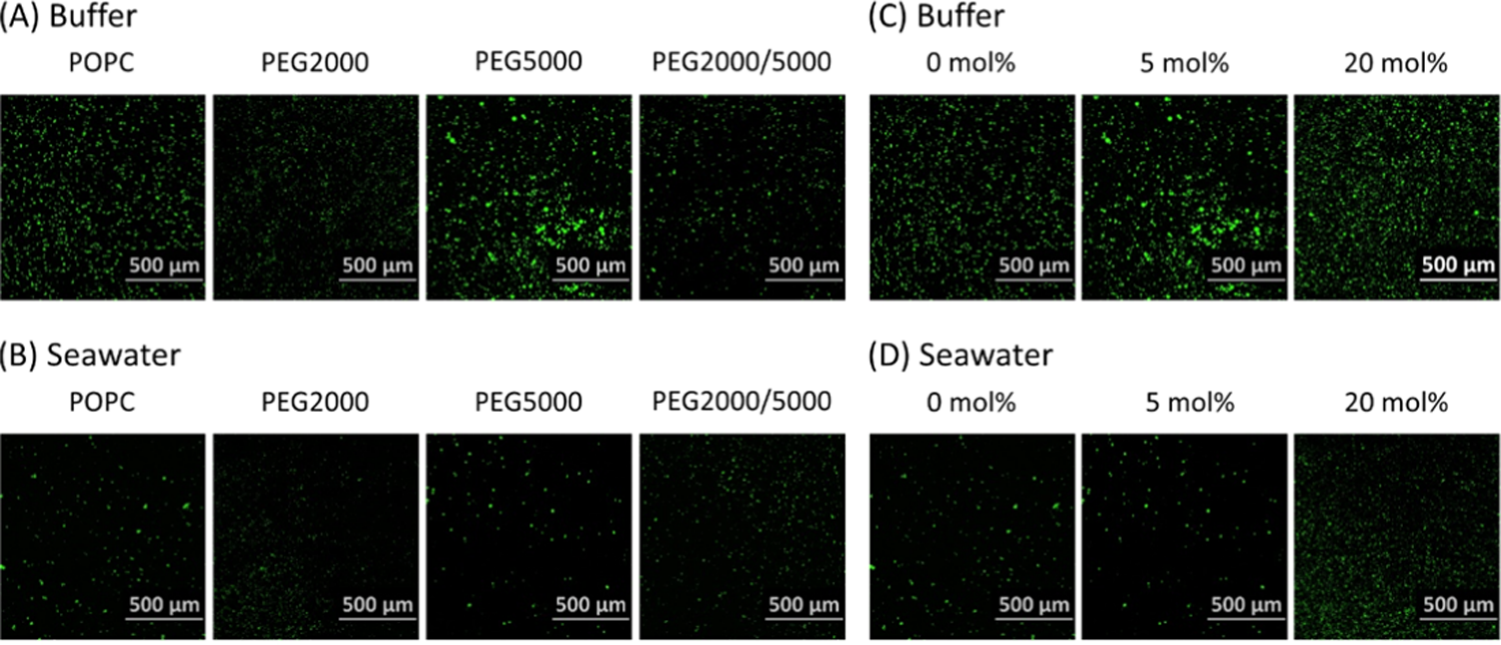
Liposomes on Day 3 with different membrane compositions. POPC, DSPE-PEG2000, DSPE-PEG5000, and a mixture of DSPE-PEG2000/DSPE-PEG5000 = 1:1 (mol/mol) (DSPE-PEG2000/5000) were prepared in (A) buffer solution and (B) natural seawater. POPC and 5 and 20 mol % DSPE-PEG5000 liposomes were prepared in (C) buffer solution and (D) natural seawater. The inner solution of the liposomes was colored green by calcein fluorescence.

**Figure 3:**
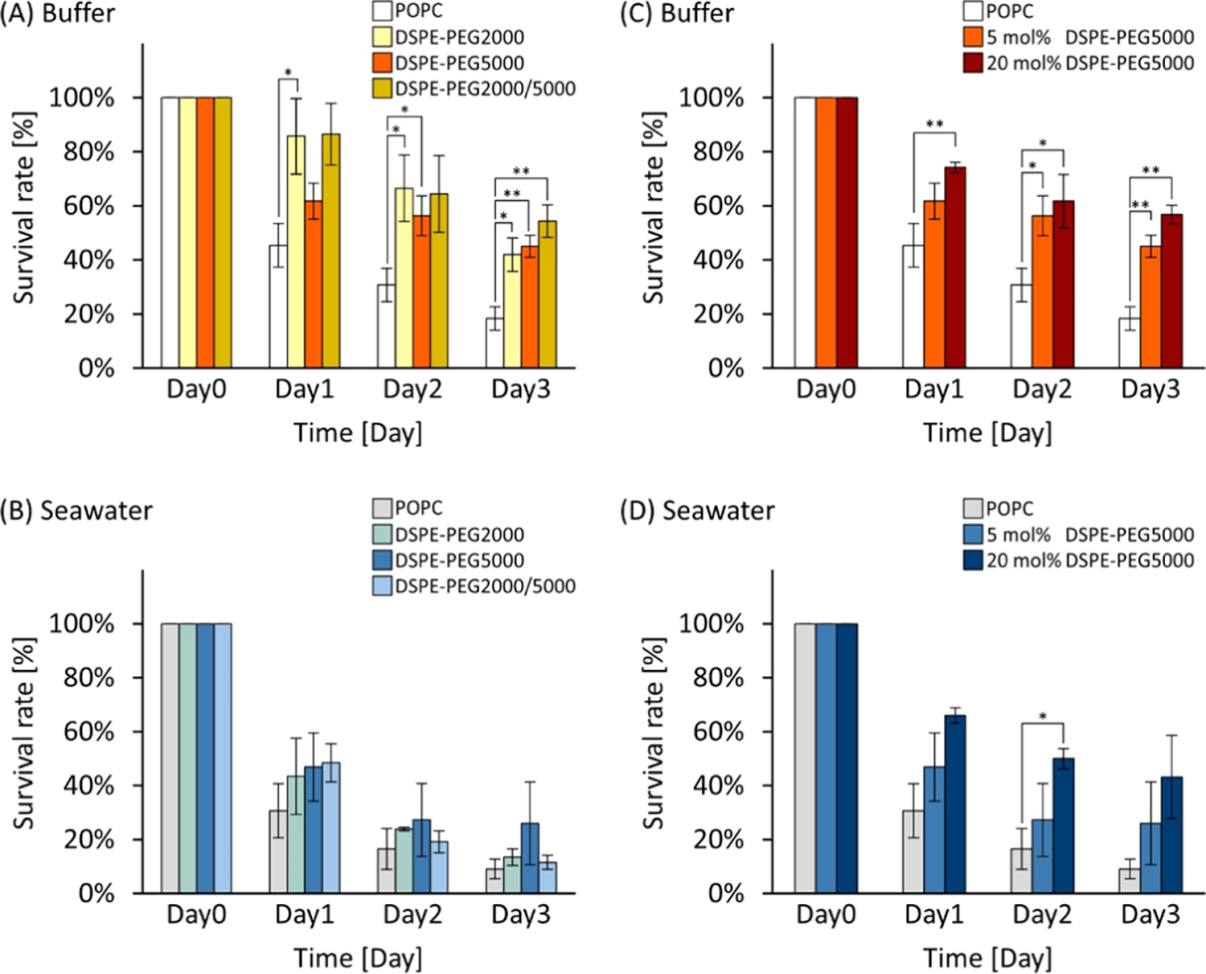
PEG properties and the survival rate. (A, B) Average survival rate of 5 mol % DSPE-PEG2000, DSPE-PEG5000, and DSPE-PEG2000/5000 in (A) buffer solution and (B) natural seawater. (C, D) Average survival rate of POPC and 5 and 20 mol % DSPE-PEG5000 liposomes in (C) buffer solution and (D) natural seawater. The horizontal axis shows the number of days after liposome preparation, and the vertical axis shows the survival rate. *p < 0.05 and **p < 0.01. Trials *N* ≥ 3 for each.

POPC or DSPE-PEG/POPC liposomes were prepared using a droplet transfer method.^31^ Initially, we expected that the addition of DSPE-PEG to the POPC membrane might reduce the productivity of liposome generation due to the long hydrophilic PEG chains. The addition of DSPE-PEG did not significantly affect productivity, even when the PEG length was changed from PEG2000 to PEG5000 (Figure S2A). However, the molar ratio of DSPE-PEG/POPC did have an impact on liposome productivity, and 30 mol % DSPE-PEG resulted in insufficient liposome generation (Figure S2B). As a result, we used the DSPE-PEG content to a maximum of 20 mol % in the subsequent experiments. Next, we investigated the temporal stability of the liposomes in both buffer solution and natural seawater from the Miura peninsula in Japan using confocal microscopy over a period of 3 days. During the period, the liposome solution was protected from light and stored at RT. Figure 2 illustrates the microscopic observations of liposomes with different lengths of the PEG chains (Figure 2A,B) and different molar ratios of DSPE-PEG/POPC (Figure 2C,D) after 3 days. Notably, the number of surviving liposomes in the seawater was significantly lower compared with the buffer. Figure 3 provides a quantitative analysis of the mean survival rate over 3 days. The data on Day 3 were used for the subsequent evaluation. Among all the membrane conditions, POPC liposomes exhibited the lowest survival rate, decreasing to approximately 18% in the buffer and 9% in the seawater on Day 3. In contrast, liposomes containing DSPE-PEG showed greater stability in both the buffer and the seawater. The length of the PEG chain had no significant effect on the stability, whereas the molar ratio affected the stability in both of them (Figure 3). In the buffer solution, the survival rate of liposomes with the 20 mol % DSPE-PEG system was approximately threefold higher than in the POPC system (on Day 3 in Figure 3C). Even though a similar difference could not be detected with significance (*p* < 0.05) in the case of seawater, the stability in the seawater was also improved by increasing the molar ratio; the mean survival rate in the 20 mol % DSPE-PEG system was fivefold higher than in the POPC system (on Day 3 in Figure 3D). The presence of DSPE-PEG seemed to be a more substantial improvement in the temporal stability within the seawater compared to that of the buffer (Figure 3C,D).

We next checked the size of the liposome because it is an important parameter and should affect the stability.^33,34^ The size distribution was then evaluated by the interquartile range (IQR) of the liposome radius. In all conditions, the IQR converged 0.7 < IQR < 3.4 μm (see also the Supporting Information Text and Figure S3), suggesting that the size did not affect the stability in this experiment. We also investigated the effect of DSPE itself, without the PEG chain, on the liposome stability because DSPE is a saturated lipid, and it should be possible to form a more rigid membrane than POPC. The survival rate of liposomes of DSPE/POPC (2:8 mol/mol) without PEG showed a similar tendency to that of POPC liposomes (Figure S4), indicating that the PEG coating mainly contributed to the liposome stability. The PEG configuration on the membrane was then estimated based on the Flory radius because the conformation of PEGs on the membrane should affect the liposome stability (see also the Supporting Information Text and Table S2). The conformations of 5 and 20 mol % DSPE-PEG were brush and dense brush, implying that these conformations of PEGs may contribute to improving the liposome stability. Since experiments up to this point were conducted for 3 days, we next examined the longer, 14-day stability. The survival rate of liposomes with 20 mol % DSPE-PEG on Day 14 was 3% even in the seawater (Figure S5). These findings indicate that incorporating DSPE-PEG into the liposomes improves their temporal stability, particularly in natural seawater, and that a molar ratio of 20 mol % DSPE-PEG offers the best results for enhancing liposome survival.

### 3.2 Liposome Stability Decreased by Cations Contained in Natural Seawater

#### 3.2.1 Monovalent and Divalent Cations Decreased Liposome Stability

**Figure 4:**
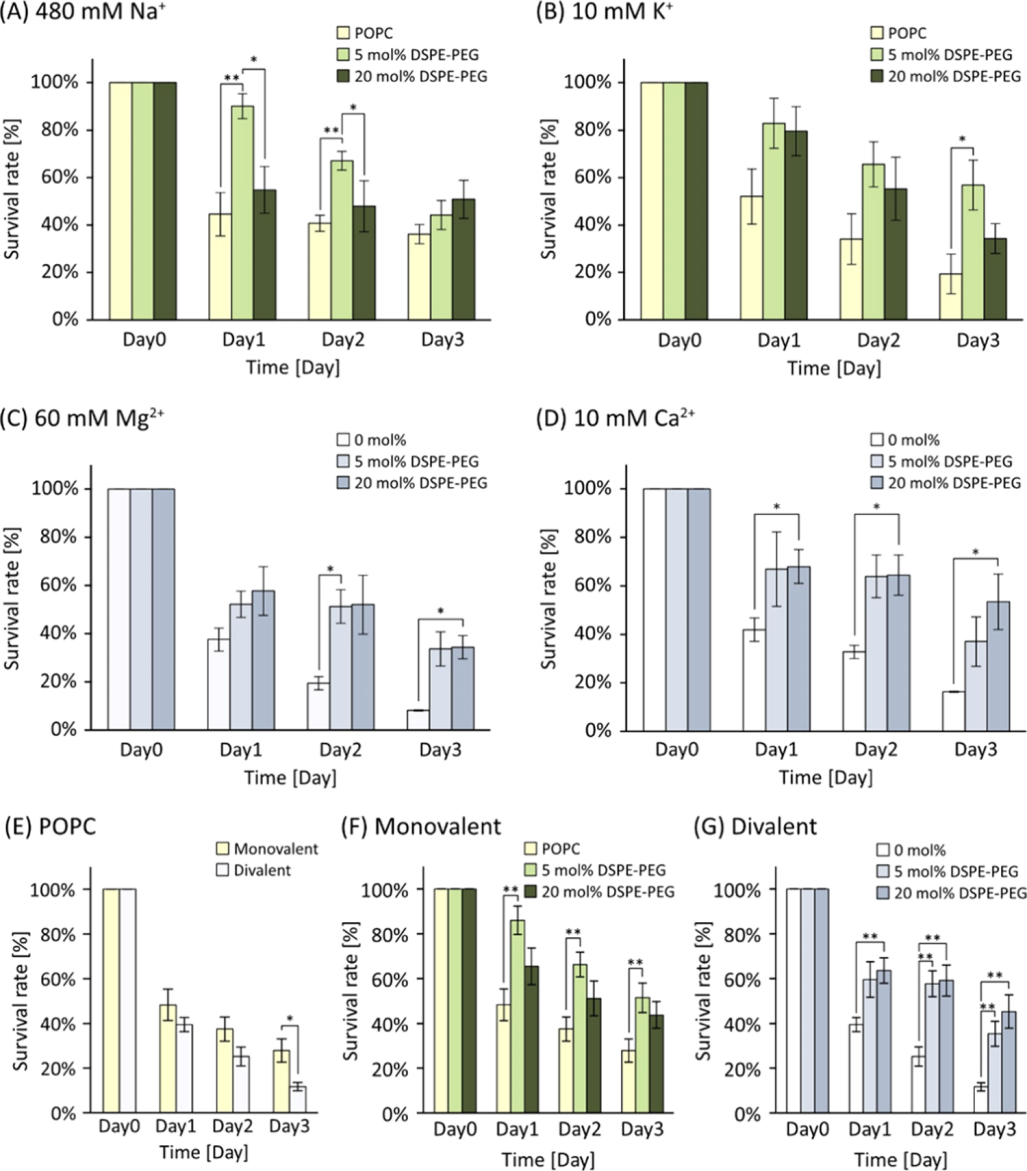
Liposome stability in four cationic solutions. Average survival rate of POPC and 5 and 20 mol % PEG liposomes in (A) 480 mM sodium ion (Na^+^), (B) 10 mM potassium ion (K^+^), (C) 60 mM magnesium ion (Mg^2+^), and (D) 10 mM calcium ion (Ca^2+^) buffers. Each ionic concentration was adjusted the same as natural seawater. (E) Average survival rate of POPC in monovalent or divalent solution. Average survival rate of POPC and 5 and 20 mol % PEG liposomes in (F) monovalent ions (Na^+^ and K^+^) and (G) divalent ions (Mg^2+^ and Ca^2+^). *p < 0.05 and **p < 0.01. Trials *N* ≥ 3 for each.

Although the stability of liposomes in natural seawater tended to be lower compared to that in the buffer solution, we observed that DSPE-PEG/POPC liposomes appeared to improve stability in both environments compared to POPC liposomes. Interestingly, the introduction of DSPE-PEG seemed to have a more effective effect on stability in the seawater. In our experiments, the buffer solution contained only glucose and tris-HCl, leading us to hypothesize that the PEG chains might mitigate interactions between cations and the lipid membrane. Natural seawater contains a higher concentration of various ions, including monovalent and divalent cations, which can interact with lipid heads and water molecules and then reduce the dipole potential of the membrane.^23−27^ To investigate the effects of cations contained in the seawater, we evaluated liposome stability in electrolyte solutions that contain one of the cations, Na^+^, K^+^, Mg^2+^, or Ca^2+^, with a similar concentration found in nature (Table 1).^20^ Notably, the order of survival rates exhibited a consistent trend even among different ion species (Na^+^ and K^+^) in the case of monovalent ions (Figure 4A,B). In the divalent cations (Ca^2+^ and Mg^2+^), the survival rate of 20 mol % DSPE-PEG liposomes was the highest (Figure 4C,D). The influence of cation valence on liposome stability was significant and is depicted in Figure 4E–G. In the case of POPC liposomes, the survival rate was substantially lower in divalent solutions compared to that in monovalent ones (Figure 4E). The presence of DSPE-PEG led to a more substantial improvement in stability within divalent solutions compared with monovalent systems (Figure 4F,G). The difference in survival rate between POPC and the most stable membrane condition on Day 3 was the largest in divalent solutions (threefold in Mg^2+^ with *p* < 0.05 and fourfold in Ca^2+^ with *p* < 0.05) than in monovalent solutions (onefold in Na^+^ with no significant difference and twofold in K^+^ with *p* < 0.05). Interestingly, the survival rate of 20 mol % DSPE-PEG liposomes in divalent solutions on Day 3 was comparable to that in monovalent ones. These insightful findings underscore the greater influence of divalent ions in reducing liposome stability while highlighting the effectiveness of DSPE-PEG introduction in enhancing stability against these ions.

#### 3.2.2 PEG Liposomes Were More Stable in High-Cationic Solutions than in Low-Cationic Ones

**Figure 5:**
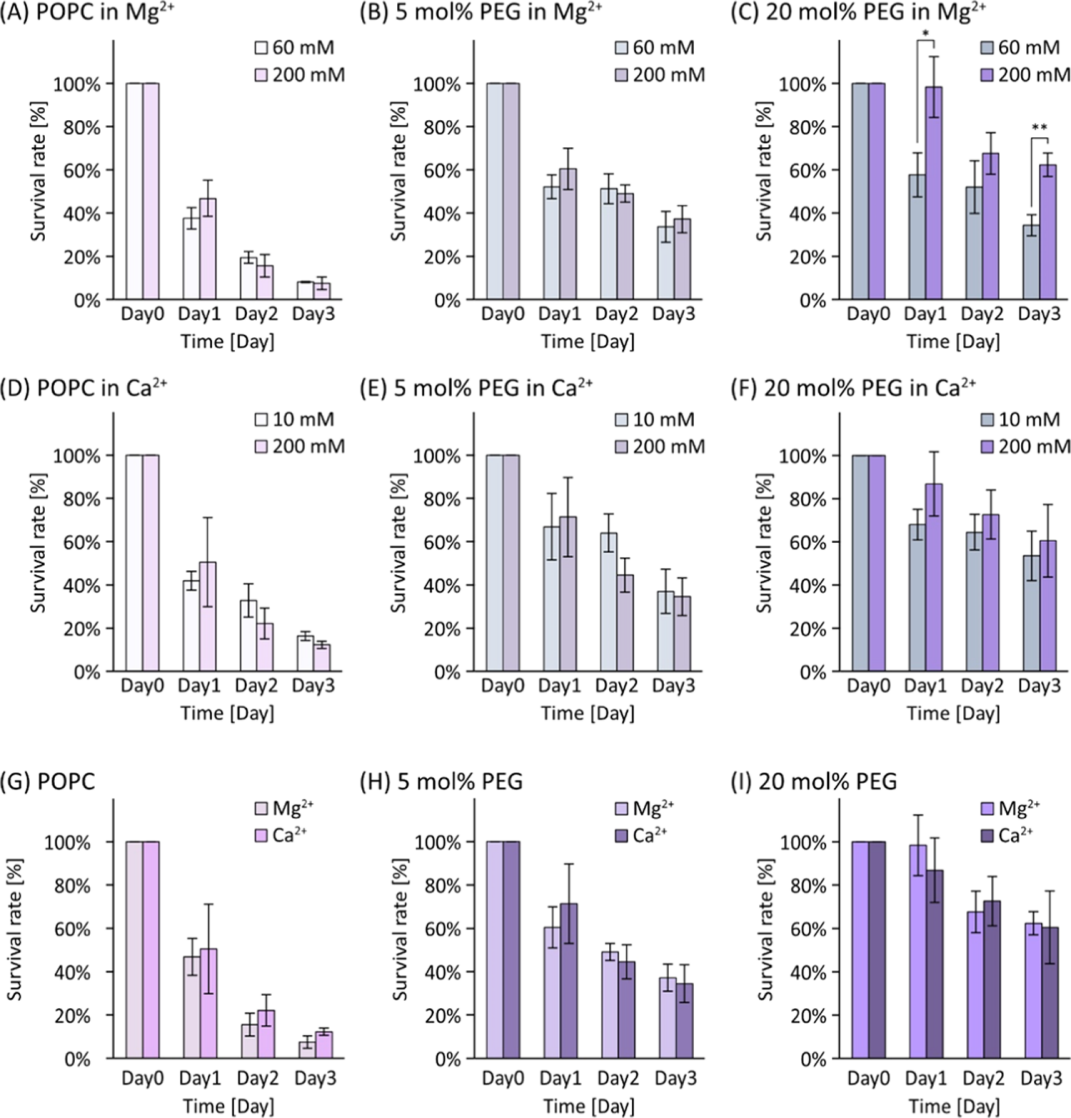
Liposome stability in high-divalent solutions. Average survival rate of POPC and 5 and 20 mol % PEG liposomes in (A–C) Mg^2+^ and (D–F) Ca^2+^. (G–I) Average survival rate of POPC and 5 and 20 mol % PEG liposomes in 200 mM divalent ions. *p < 0.05 and **p < 0.01. Trials *N* ≥ 3 for each.

To study further the effect of divalent ions, we conducted stability tests of liposomes under identical concentrations of Mg^2+^ and Ca^2+^ solutions (200 mM) as depicted in Figure 5. The presence of DSPE-PEG enhanced the stability: the survival rate of 5 mol % DSPE-PEG liposomes closely resembled that of low-ionic solutions, while 20 mol % DSPE-PEG liposomes exhibited even higher stability than that of low-ionic conditions (Figure 5B,C,E,F). The difference between POPC and 20 mol % DSPE-PEG liposomes was striking, showing an eightfold increase in Mg^2+^ with *p* < 0.01 and an impressive fivefold increase in Ca^2+^ with *p* < 0.05. Contrary to our initial expectations, there did not emerge a significant difference between the two cations (Figure 5G–I).

## 4 Discussion

Here, we attempted to improve the temporal stability of the liposomes with a DSPE-PEG/POPC membrane in natural seawater. The stability of the liposomes improved with increasing the DSPE-PEG ratio (Figure 3C,D), and the stability was affected by the types of cations contained in the seawater (Figure 4E–G). In the case of the liposome consisting of the POPC membrane itself, the stability of the liposome tended to decrease with higher concentrations of cations (Figure 5A,D). In contrast, the stability was improved with the introduction of 20 mol % DSPE-PEG into the POPC membrane even in the higher ionic solutions (Figure 5C,F). We further found that the introduction of the DSPE-PEG improved the stability against the divalent cations, although the divalent ions such as Mg^2+^ and Ca^2+^ remarkably decreased the stability of the POPC liposome (Figure 5).

**Figure 6:**
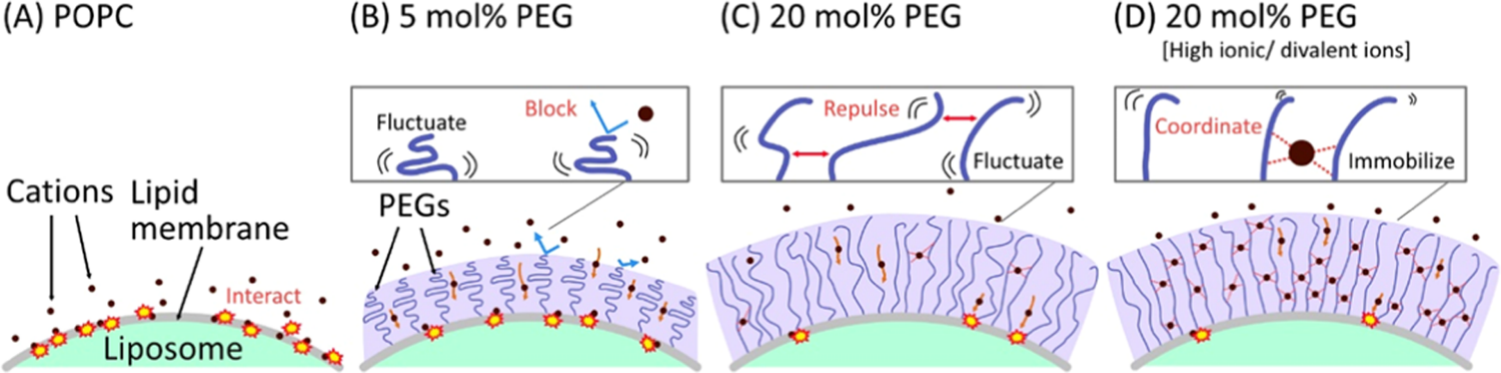
Schematic diagram of liposome stabilization/destabilization. Liposome stabilization of (A) POPC, (B) 5 mol % DSPE-PEG, (C) 20 mol % DSPE-PEG with low-affinity ions or in low-ionic solution, and (D) 20 mol % DSPE-PEG in high-ionic solution.

Based on the results, the effects of the DSPE-PEG introduction are attempted to be explained based on the PEG chain configuration as shown in Figure 6. First, cations could decrease the stability of the POPC liposome by interacting with the membranes (Figure 6A).^23−27^ The divalent ions, which have larger hydrated radii than those of the monovalent ions, are considered to have a stronger binding ability and a slower exchange rate with oxygen moieties (Table S3).^35−38^ The ions should alter the membrane properties by binding to lipid oxygens, and the binding strengths could affect the disruption of lipid alignment, thus destabilizing liposomes. Next, DSPE-PEGs improve liposome stability by forming a shield-like layer that prevents the cations from accessing deep inside the PEG layer as previously reported.^16−18^ The effects were enhanced with increasing DSPE-PEG ratio and in the case of the high-cationic solution (Figure 6B–D). Since the formation of PEG–cation complexes has been experimentally and theoretically studied,^38−40^ the PEG–cation complexes should also form on the surface of liposomes, resulting in the formation of a rigid shield-like layer with the PEG–cation complex.

For the actual implementation of the liposome-based molecular robot in natural seawater, several uncertainties remain. Even if ions are the main components, natural seawater also contains a wide variety of organic components such as amino acids^21^ and fatty acids,^22^ and they have the potential to interact with the membrane, change the properties, and affect the liposome stability. For instance, serine, which is an amino acid found in natural seawater,^21^ can interact with the lipid membrane and change the membrane rigidity.^41^ Other environmental conditions such as pH and temperature should also be considered in further investigation.

## 5 Conclusions

In conclusion, we discussed the membrane composition at which liposomes with PEG-lipid are stable in natural seawater. The liposome stability was evaluated by a microscopic observation. It turned out that the high-density PEG liposomes were the most stable in natural seawater for 3 days of observation, and some of them can live for 14 days. Factors for liposome destabilization were specified using various ionic solutions, which indicates that the divalent ions caused the destabilization. To date, liposome stability has been mainly investigated and discussed in the medical field.^42−44^ In this study, liposome stability in natural seawater was evaluated for future environmental applications. Our study can contribute to fabricating liposomal molecular robots for employing various natural environments.

## Supporting Information Available

Membrane composition for evaluation of liposome productivity, theoretical PEG conformation, physical properties of the cations, evaluation processes of liposomes, liposome productivity, size distribution of the liposome, correlation between the lipid type and liposome stability, and liposome stability on Day 14 (PDF)

## Abbreviations

PEG: poly(ethylene glycol)
DSPE-PEG: *N*-(methylpolyoxyethylene oxycarbonyl)-1,2-distearoyl-sn-glycero-3-phosphoethanolamine sodium salt
POPC: 1-palmitoyl-2-oleoyl-glycero-3-phosphocholine
DOPG: 1,2-dioleoyl-*sn*-glycero-3-phospho-(1′-rac-glycerol) (sodium salt)
rhodamine PE: 1,2-dioleoyl-*sn*-glycero-3-phosphoethanolamine-*N*-(lissamine rhodamine B sulfonyl) (ammonium salt)
W/O: water–oil
BSA: bovine serum albumin
RT: room temperature
