# Supplementary material for: Long-Term Stable Liposome Modified by PEG-Lipid in Natural Seawater
Source: ACS Omega. 2024 Feb 22;9(9):10958–66. doi: 10.1021/acsomega.3c10346 (PMC10918668; doi:10.1021/acsomega.3c10346)
Supplement: Supplementary file 1 — ao3c10346_si_001.pdf [file ao3c10346_si_001.pdf]

Supporting Information

Long-Term Stable Liposome Modified by PEG-lipid in Natural Seawater

Kayano Izumi[1], Jiajue Ji[1], Keiichoro Koiwai[2], and Ryuji Kawano[1*]

[1] *Department of Biotechnology and Life Science, Tokyo University of Agriculture and Technology, Tokyo, 184-8588, Japan*

[2] *Laboratory of Genome Science, Tokyo University of Marine Science and Technology, Tokyo, 108-8477, Japan*

*Corresponding author

Osmotic measurement of natural seawater

The osmolality of natural seawater was measured experimentally using an osmometer (VAPRO® Vapor Pressure Osmometer Model 560, ELI Tech Group Inc., USA). We sampled the natural seawater from the Misaki peninsula. The osmolality was 993.3 ± 2.0 mmol/kg, which was assumed to contain approximately 1000 mM of chemicals (Trials N= 8). In our system using an inverted confocal microscope, the liposome needed to be near the bottom of the chamber, to close the objective lens. To sink the liposomes while maintaining the isotonic conditions, we used sucrose as the inner component and glucose as the outer component. All measurements were performed at room temperature.

Selection of PEG lipids

The structural difference of PEG chains can be classified into length and ratio. For length, we used PEG2000 and PEG5000. To our knowledge, the stability of cell-sized liposomes by PEG coating has hardly been reported, but the two PEG lipids have been used in medicine to prepare submicron-sized liposomes because the length is effective both to form liposomes without aggregation[1] and to prevent macromolecules such as plasma proteins.[2-4] For the ratio, we attempted 0 (POPC), 1, 5, 20, 30, 40, and 50 mol% DSPE-PEG5000/POPC liposomes and compared the productivity or survival rate. We also prepared the PEG mixture of DSPE-PEG2000: DSPE-PEG5000 = 1: 1 (mol/mol) (DSPE-PEG2000/5000) with the expectation of forming a bumpy branch-like uneven lateral distribution of PEG chains on the membrane.

Membrane composition

Table S1. Membrane composition for evaluation of liposome productivity. [a]

label		membrane composition		notes
		DSPE-PEG [%]	POPC [%]	
POPC		0	100	lipid only
5 mol%	DSPE-PEG2000	5	95	-
5 mol%	DSPE-PEG2000/5000	Total 5	95	the mixture of DSPE-PEG2000: DSPE-PEG5000=1:1 (mol/mol)
5 mol%	DSPE-PEG5000	5	95	-
1 mol%	DSPE-PEG5000	1	99	-
20 mol%	DSPE-PEG5000	20	80	also prepared by fivefold lipids
30 mol%	DSPE-PEG5000	30	70	also prepared by 100-fold lipids
40 mol%	DSPE-PEG5000	40	60	also prepared by 100-fold lipids
50 mol%	DSPE-PEG5000	50	50	-

a. Liposome productivity was confirmed for various membrane compositions. Since the productivity varied widely at higher ratios of DSPE-PEGs to the initial amount of lipids, liposomes were prepared with fivefold (20 mol %) and 100-fold (30 and 40 mol %) amounts of lipids to confirm whether the initial amount of lipids was too small to form liposomes or difficult to form liposomes at the compositions. For each composition, +1 mol% DOPG was added to avoid liposomes adhering to each other, and +0.1 mol% Rhodamine PE was added to easily detect liposomes.

PEG configuration

The PEG configuration was quantitatively estimated as previously proposed.[5, 6] The degree of polymerization (N) of PEG2000 and PEG5000 was 45 and 114, respectively, and the size (a) was defined as 0.39 nm.[5] The Flory radius (R_F) was calculated using the following equation:

$$R_F \approx aN^{3/5} \qquad \text{S1}$$

The Flory radius (R_F) of PEG2000/5000 was assumed by defining N as 79.5, which is the average of PEG2000 and PEG5000. The distance between the PEG chains (D) and the length/ thickness of the PEGs (L) were also calculated using the following equations:

$$D = \sqrt{A_{lipid}/m} \qquad \text{S2}$$

$$L = aN(a/D)^{2/3} \qquad \text{S3}$$

The area per lipid (A_{lipid}) were defined as 0.48- 0.67 nm^2, the m is the mole fraction of PEG, and the PEG conformation was classified when $D > R_F$ as mushroom, $D < R_F$ as brush, and $L > 2R_F$ as dense brush [5, 6].

Table S2. Theoretical PEG conformation. [a]

label		conformation
POPC		-
5 mol%	PEG2000	Brush
5 mol%	PEG2000/5000	Brush
1 mol%	PEG5000	Mushroom
5 mol%	PEG5000	Brush
20 mol%	PEG5000	Dense brush
30 mol%	PEG5000	Dense brush
40 mol%	PEG5000	Dense brush
50 mol%	PEG5000	Dense brush

a. Calculated PEG conformations for all membrane compositions. The PEG conformation was calculated to estimate the relation between liposome stabilization and the PEG ratio.

Liposome size

A size effect on the liposome stability should be considered when there was a difference of tens of microns depending on the membrane compositions. We evaluated the size by using the liposome radius. Since the distributions of liposome radius did not follow the Gaussian distribution, we estimated the distribution using the interquartile range (IQR). The IQR can be expressed by the following equation:

$$IQR = third\ quartile - first\ quartile \tag{S4}$$

Table S3. The physical properties of the cations. [a]

cation	hydrated radius [Å] [7]	exchange rate* $[s^{-1}]$[8, 9]	coordination numbers [9-11]	crystal radius [Å] [7]
Na^+	2.8	$10^9 \sim 10^{10}$	4 ~ 8	1.1
K^+	2.7	$10^9 \sim 10^{10}$	6 ~ 8	1.4
Mg^{2+}	3.6	10^6	6	0.7
Ca^{2+}	3.4	$10^8 \sim 10^9$	8	1.1

a. The differences in the properties caused the differences in the ability to bind to oxygen moieties. The approximate values were assumed based on previous studies. *The exchange rate constant for water molecules.

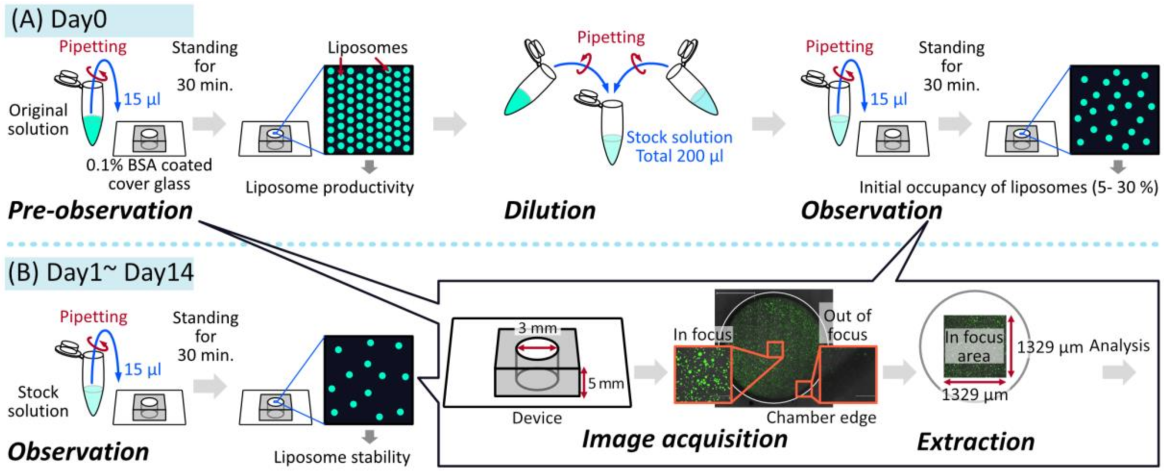

Figure S1. The evaluation processes of liposomes. Observation (A) on Day0 and (B) on Day1~Day14.We first obtained the whole image of the chamber. Since the horizontal coordination was slightly different between the center and the edge of the chamber, we extracted a 1329 x 1329 μm^2 square unit area for analysis.

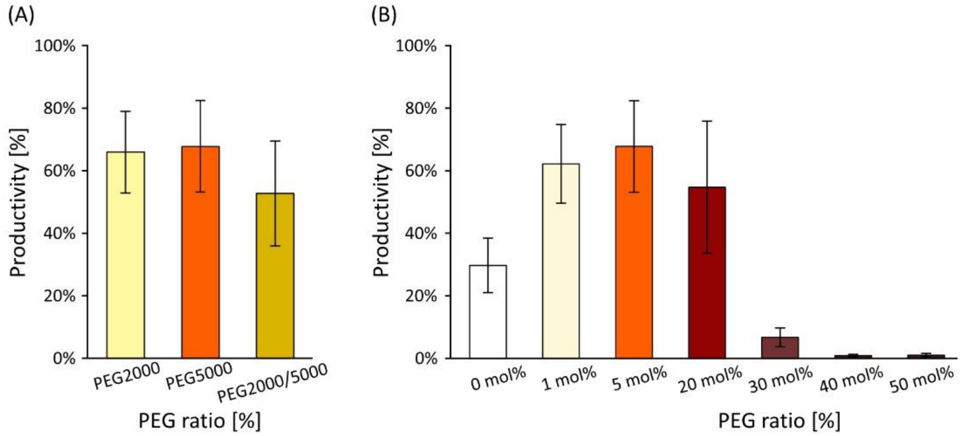

Figure S2. The liposome productivity. The average productivity of liposomes with (A) the different DSPE-PEG length contained at 5mol% and (B) the different ratio of DSPE-PEG5000 in buffer. Trials N≥ 3 for each**.**

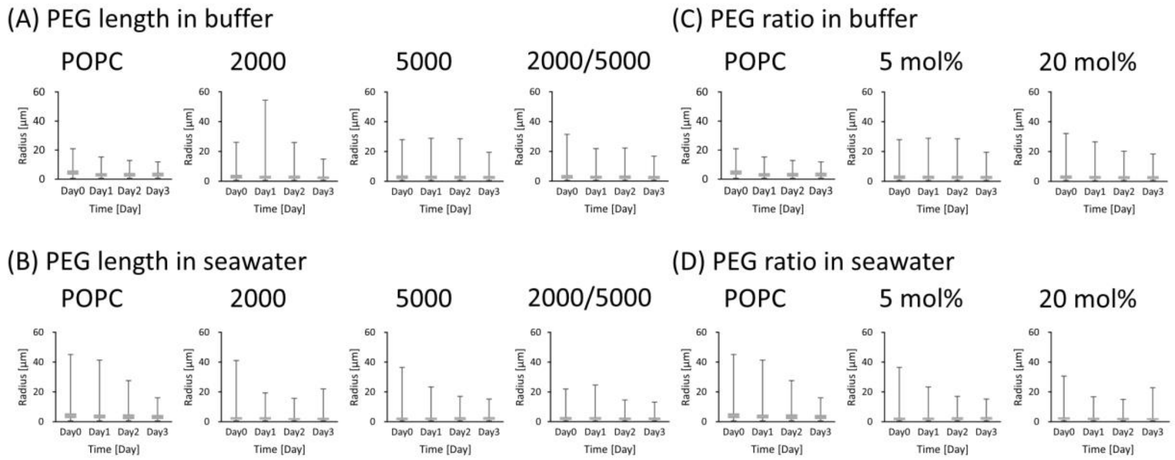

Figure S3. The size distribution of the liposome. Trials N≥ 3 for each.

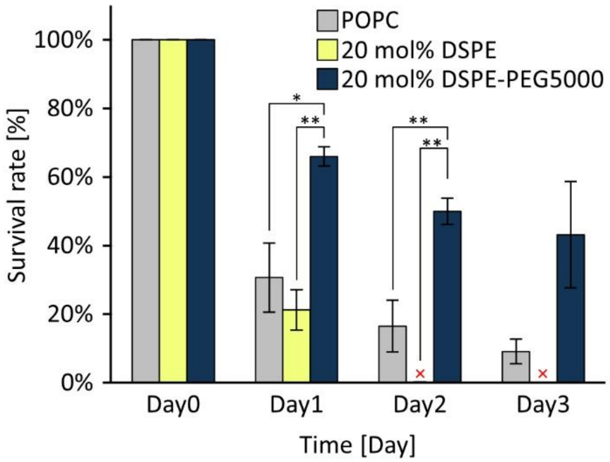

Figure S4. Correlation between the lipid type and liposome stability. The survival rate in natural seawater. The red symbols ‘x’ indicate that the survival rate was less than 1%. *$p < 0.05$ and**$p < 0.01$. Trials N≥ 3 for each.

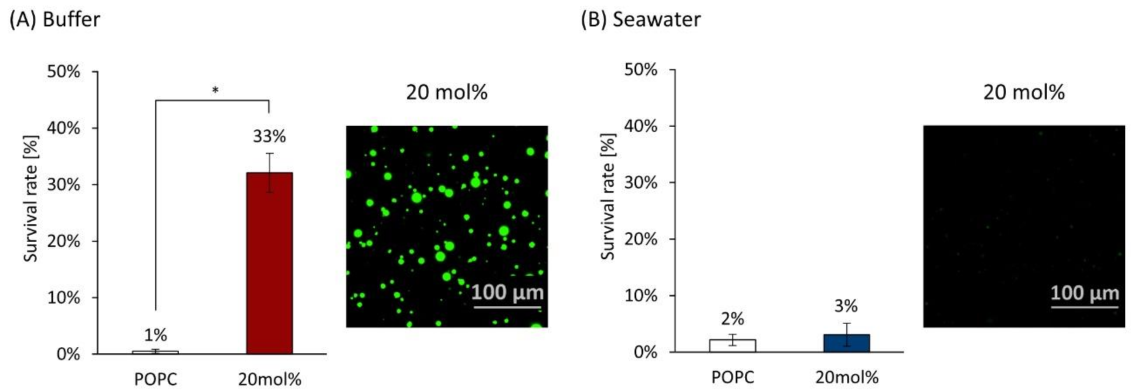

Figure S5. Liposome stability on Day 14. The survival rate (A) in buffer solution and (B) in natural seawater. *$p < 0.05$. Trials N≥3 for each.

REFERENCES

(1) Rovira-Bru, M.; Thompson, D. H.; Szleifer, I. Size and Structure of Spontaneously Forming Liposomes in Lipid/PEG-Lipid Mixtures. *Biophysical Journal* **2002**, *83*, 2419–2439. DOI: 10.1016/S0006-3495(02)75255-7.
(2) Ashok, B.; Arleth, L.; Hjelm, R. P.; Rubinstein, I.; Onyuksel, H. In vitro characterization of PEGylated phospholipid micelles for improved drug solubilization: effects of PEG chain length and PC incorporation. *J Pharm Sci* **2004**, *93* (10), 2476-2487. DOI: 10.1002/jps.20150.
(3) Rydberg, H. A.; Yanez Arteta, M.; Berg, S.; Lindfors, L.; Sigfridsson, K. Probing adsorption of DSPE-PEG2000 and DSPE-PEG5000 to the surface of felodipine and griseofulvin nanocrystals. *Int J Pharm* **2016**, *510* (1), 232-239. DOI: 10.1016/j.ijpharm.2016.06.046.
(4) Sou, K.; Endo, T.; Takeoka, S.; Tsuchida, E. Poly(ethylene glycol)-Modification of the Phospholipid Vesicles by Using the Spontaneous Incorporation of Poly(ethylene glycol)-Lipid into the Vesicles. *Bioconjugate Chemistry* **2000**, *11* (3), 372-379. DOI: 10.1021/bc990135y
(5) Marsh, D.; Bartucci, R.; Sportelli, L. Lipid membranes with grafted polymers: physicochemical aspects. *Biochimica et Biophysica Acta (BBA) - Biomembranes* **2003**, *1615* (1-2), 33-59. DOI: 10.1016/s0005-2736(03)00197-4.
(6) Abou-Saleh, R. H.; Swain, M.; Evans, S. D.; Thomson, N. H. Poly(ethylene glycol) lipid-shelled microbubbles: abundance, stability, and mechanical properties. *Langmuir* **2014**, *30* (19), 5557-5563. DOI: 10.1021/la404804u.
(7) Tansel, B.; Sager, J.; Rector, T.; Garland, J.; Strayer, R. F.; Levine, L.; Roberts, M.; Hummerick, M.; Bauer, J. Significance of hydrated radius and hydration shells on ionic permeability during nanofiltration in dead end and cross flow modes. *Separation and Purification Technology* **2006**, *51* (1), 40-47. DOI: 10.1016/j.seppur.2005.12.020.
(8) Helm, L.; Merbach, A. E. Inorganic and Bioinorganic Solvent Exchange Mechanisms. *Chemical Reviews* **2005**, *105* (6), 1923−1959. DOI: 10.1021/cr030726o.
(9) Umebayashi, Y.; Yamaguchi, T. *Electrolyte solution (Denkaishitsu youeki, in Japanese)*; Maruzen, 2013.
(10) Long, M. P.; Alland, S.; Martin, M. E.; Isborn, C. M. Molecular dynamics simulations of alkaline earth metal ions binding to DNA reveal ion size and hydration effects. *Phys Chem Chem Phys* **2020**, *22* (10), 5584-5596. DOI: 10.1039/c9cp06844a.
(11) Babu, C. S.; Lim, C. Theory of Ionic Hydration: Insights from Molecular Dynamics Simulations and Experiment. *The Journal of Physical Chemistry B* **1999**, *103* (37), 7958–7968. DOI: 10.1021/jp9921912.
